# How can heatstroke damage the brain? A mini review

**DOI:** 10.3389/fnins.2024.1437216

**Published:** 2024-10-10

**Authors:** Kazuhiro Yoneda, Sanae Hosomi, Hiroshi Ito, Yuki Togami, Sayaka Oda, Hisatake Matsumoto, Junya Shimazaki, Hiroshi Ogura, Jun Oda

**Affiliations:** ^1^Department of Traumatology and Acute Critical Medicine, Osaka University Graduate School of Medicine, Suita, Japan; ^2^Laboratory of Human Immunology (Single Cell Genomics), WPI Osaka University Immunology Research Center, Osaka University, Osaka, Japan

**Keywords:** heatstroke, blood–brain barrier, central nervous system injury, genetic analyses, diagnosis of heat-related illnesses

## Abstract

Record-breaking heat waves over the past 20 years have led to a global increase in heat-related deaths, including heatstroke. Heat-related illnesses occur when the body cannot adapt to the elevated temperatures in the environment, leading to various symptoms. In severe situations, such as heatstroke, the body temperature can rise above 40°C, leading to significant injury to body systems, with particular susceptibility of the central nervous system (CNS). Neuroimaging studies conducted months or years after a heatstroke have revealed cellular damage in the cerebellum and other brain regions, including the hippocampus, midbrain, and thalamus, with the potential for long-term neurological complications in survivors of a heatstroke. This mini review aimed to describe the mechanisms and pathways underlying the development of brain injury induced by heatstroke and identify diagnostic imaging tools and biomarkers for injury to the CNS due to a heatstroke.

## 1 Introduction

Heat-related illnesses are a significant global health concern, affecting a substantial number of individuals annually. Escalating global temperatures due to climate change have intensified the frequency and severity of heat-related illnesses (Robine et al., 2008). These range from mild conditions, such as heat-induced syncope and cramps, to severe conditions, such as heat exhaustion and life-threatening heatstroke (Bouchama and Knochel, 2002; Savioli et al., 2022; Barletta et al., 2024). Heatstroke is a medical emergency associated with a high mortality rate of 20–60% (Bouchama et al., 2022). The pathophysiology of acute severe heatstroke is complex and may involve multiple organ systems, leading to complications such as rhabdomyolysis, disseminated intravascular coagulation (DIC), acute renal failure, liver damage, acute respiratory distress syndrome, electrolyte imbalances, and neurological complications (Dematte et al., 1998; Pechlaner et al., 2002; Bouchama and Knochel, 2002; Lawton et al., 2019). Neurological complications include cerebellar ataxia, cognitive impairment, dysphagia, and aphasia (Lawton et al., 2019).

This mini review aimed to highlight the importance of understanding the mechanisms and pathways underlying brain injury induced by heatstroke and identifying diagnostic imaging tools and biomarkers crucial for developing targeted management strategies. Most recent advancements in this field are summarized and discussed.

## 2 Review

### 2.1 Heatstroke: a growing concern in a warming world

Heatstroke, a severe form of heat-related illness, is becoming increasingly common owing to rising global temperatures (Bouchama and Knochel, 2002). Heatstroke is characterized by a core body temperature exceeding 40°C (104°F), leading to central nervous system (CNS) dysfunction (Becker and Stewart, 2011; Bouchama et al., 2022). Heatstroke can be classified into two types: classical (non-exertional) heatstroke, which typically affects vulnerable populations such as older adults and infants, and exertional heatstroke, which is more common among young, healthy individuals engaged in physical activities (Gaudio and Grissom, 2016; Hifumi et al., 2018). According to the World Health Organization, approximately 166,000 individuals succumbed to heatstroke between 1998 and 2017, with the 2003 European heat wave alone resulting in an estimated 70,000 deaths (Robine et al., 2008). The occurrence of heatstroke varies worldwide, and factors that increase the risk of heatstroke are geographical locations with high temperatures, limited access to cooling resources, and inadequate public health infrastructure (Rublee et al., 2021) (Supplementary Table S1).

### 2.2 Classification and pathology of heatstroke

Heat-related illnesses encompass a spectrum of conditions resulting from the body’s inability to adequately adapt to the stresses imposed by high-temperature environments. Historically, heat-related illnesses have been classified according to the presence or absence of exertion, symptoms, or temperature; however, the lack of a globally uniform definition has made comparisons difficult. In Japan, a classification stratified by severity, as shown in Supplementary Table S2, has become widespread (Hifumi et al., 2018). Under normal physiological conditions, an increase in core body temperature activates the hypothalamic thermoregulatory center, inducing adaptive mechanisms, such as sweating and increased skin blood flow, to dissipate excess heat. Dilation of blood vessels facilitates the distribution of cooler blood throughout the body. However, dehydration can reduce the overall blood volume, leading to diminished cerebral perfusion and symptoms, such as headaches and altered consciousness, known as heat syncope. Concurrently, the process of sweating, while aiding thermoregulation through cooling by evaporation, also results in remarkable electrolyte losses, particularly sodium (Na+), leading to muscle cramps and spasms. Accordingly, the initial management of heat-related illness generally includes the use of oral rehydration solutions and/or moving the individual to a cooler environment. Impacts of dehydration and fatigue can exacerbate the condition, with progression to second-degree heatstroke, also known as heat exhaustion, which necessitates medical intervention. Further progression of the condition results in the cessation of sweating and, if unchecked, increase in core temperature, culminating in third-degree heatstroke, characterized by widespread organ damage, including endothelial injury.

### 2.3 Pathophysiology of heatstroke and mechanism of body temperature elevation

The pathophysiology of heatstroke is characterized by the failure of thermoregulation, leading to systemic inflammation, coagulopathy, and multi-organ dysfunction, encompassing acute renal failure, liver damage, and acute respiratory distress syndrome (Hifumi et al., 2018; Iba et al., 2022). Systemic effects of thermoregulation failure are exacerbated by profound dehydration-induced circulatory failure, direct thermal damage, and hyper-cytokinemia (Hifumi et al., 2018; Bouchama et al., 2022). The interplay between inflammation and coagulation plays a pivotal role in propagating organ dysfunction, with heat-induced damage triggering a cascade of cellular and molecular responses that exacerbate vascular permeability and coagulopathy, leading to DIC and multi-organ failure. When damaged, cells release damage-associated molecular proteins (DAMPs), such as high mobility group box (HMGB)-1, histones, and DNA. DAMPs activate inflammasomes through pattern recognition receptors, causing an inflammatory response through the release of cytokines (Iba et al., 2022).

Similar to sepsis and trauma, heatstroke causes a systemic inflammatory reaction syndrome (SIRS) following hyperthermia (Bouchama et al., 2022).

Heat-induced SIRS results from a failure of thermoregulation and elevation in core temperature, which directly damages endothelial cells, leukocytes, and epithelial cells. Cell damage causes release of DAMPs, such as HMGB-1, histones, and DNA, which bind to pattern recognition receptors (PPRs), such as toll-like receptors and inflammasomes. It progressively worsens vascular permeability and coagulopathy, leading to DIC and multiple organ failure (Epstein and Yanovich, 2019).

### 2.4 Heatstroke damages various areas in the brain (Table 1)

**Table 1:** Brain damage and heatstroke.

Damaged lesion	Symptoms	Stage	Species	Findings	Mechanism of injury	Reference
Hypothalamus	Elevated body temperature by deranged thermoregulation	Within 24 h	Human	Edema, hemorrhage in the hypothalamus.	Thermal denaturation due to increased body temperature. Neuroinflammation due to inflammatory cytokines. Cellular edema due to increased BBB permeability.	Malamud et al. (1946), Bouchama and Knochel (2002), and Leon and Helwig (2010)
			Rat	Accumulation of iNOS in the thalamus by immunohistochemistry. Elevated MPO, TNFα, and IL-1 Elevated albumin, GFAP, and water content.		Hsiao et al. (2007), Liu et al. (2009), Kiyatkin and Sharma (2009), and Tai et al. (2021)
Cerebellum	Ataxia, balance disorders, dizziness	Within 24 h	Human	Signal changes on MRI, cerebellar atrophy on CT. Increased HSP70 expression in the cerebellum on pathological autopsy.	Increased inflammatory response. Diffuse loss of Purkinje cells.	Malamud et al. (1946), Van Stavern et al. (2000), McLaughlin et al. (2003), Leon and Helwig (2010), Walter and Carraretto (2016), and Jung et al. (2017)
			Rabbit	Sustained up-regulation of the HSP70 gene and subsequent accumulation of HSP70 mRNA.		D’Souza et al. (1998)
Cerebral cortex	Cognitive impairment, impaired consciousness	More than 24 h	Human	Edema, congestion, degenerative changes in the cerebral cortex neurons	Hemorrhages, Neuroinflammation by inflammatory cytokines. Oxidative stress and mitochondrial dysfunction.	Malamud et al. (1946), Bouchama and Knochel (2002), and Walter and Carraretto (2016)
			Mice	Down-regulation of mitochondrial outer membrane proteins Tomm40 and mitochondrial membrane permeability transition pore. Elevated GFAP, Iba-1, NFκB and COX-2		Sharma et al. (1991), Kiyatkin and Sharma (2009), and Fang et al. (2024)
Hippocampus	Memory deficit	More than 5 days	Human	Inflammation, edema of hippocampus signal change of MRI after day5	Neuroinflammation and apoptosis. Changes of hippocampal neurogenesis.	Sudhakar and Al-Hashimi (2007), Leon and Helwig (2010), Zhu et al. (2023), and Mahajan and Schucany (2008)
			Rat	Elevated Caspase-3, Fluoro Jade-C Elevated BDNF,IL-1β, IL-6, NGF and TNF-α		Chauhan et al. (2021)

BDNF, brain-derived neurotrophic factor; COX, Cyclooxygenase; CT, computed tomography; GFAP, glial fibrillary acidic protein; HSP, heat shock protein; IL, interleukin; iNOS, inducible nitric oxide synthase; MPO, myeloperoxidase; MRI, magnetic resonance imaging; NGF, nerve growth factor; TNF, tumor necrosis factor.

The hypothalamus is considered the thermoregulatory center of the body, but it is also vulnerable to heat stress. Tai et al. revealed that myeloperoxidase, tumor necrosis factor (TNF)-*α*, and interleukin (IL)-1 are elevated in the hypothalamus in a rat heatstroke model (Tai et al., 2021). Hsiao et al. demonstrated through immunohistochemistry that iNOS accumulates in the hypothalamus in rat heatstroke models (Hsiao et al., 2007). Immunohistochemistry showed considerable increases in GFAP, Iba1, NF-κB, and COX-2 (Chauhan et al., 2017). Heatstroke has been confirmed to damage nerve cells and even induce inflammation (Liu et al., 2009).

In the brain, the cerebellum is deemed the most vulnerable tissue to heat stress and is among the first areas to show signs of damage (Bazille et al., 2005). In patients who died within 24 h of heatstroke, edema had already begun in the Purkinje layer, and the number of Purkinje cells had decreased. Malamud et al. (1946) reported that by the third day, almost no Purkinje cells remained, having undergone coagulation. Li et al. (2015), in a brain MRI study, showed that fractional anisotropy was markedly higher in the cerebellum of patients with heatstroke than that of healthy individuals. Bazille et al. (2005) also showed that Purkinje cells were gradually lost in the cerebellum, accompanied by HSP70 expression in the pathological gyrus and cerebellum of three patients who had died from heatstroke. Additionally, in experiments with heatstroke rabbits, persistent upregulation of the HSP70 gene and subsequent accumulation of HSP70 mRNA have been reported (D’Souza et al., 1998). There are case reports of dizziness and nystagmus as cerebellar symptoms 1 week after the onset of heatstroke (Van Stavern et al., 2000; Jung et al., 2017).

Changes in the cerebral cortex begin to appear after 24 h. According to the pathological autopsy results of Malamud et al. (1946), no significant changes occurred up to 18 h; however, after 24 h, the number of neurons decreased and glia began to proliferate. In a juvenile rat heatstroke model by Sharma et al. (1991), neurons in the cerebral cortex darkened and astrocytes swelled. At the microstructural level, microvessels collapsed, and perivascular edema, cavitation, and synaptic damage were observed. When the cFos protein was examined to evaluate the localization of brain damage in a mouse heatstroke model, the most significant damage was observed in the anterior cingulate cortex of the cerebral cortex. In addition, a multi-omics analysis of the cerebral cortex conducted in the same study revealed that considerable changes occurred in pathways related to neurotransmission, mitochondrial dysfunction, and oxidative stress (Fang et al., 2024).

Reports reveal that MRI signals in the hippocampus begin to show changes from approximately the fifth day (Sudhakar and Al-Hashimi 2007; Mahajan and Schucany, 2008). Zhu et al. (2023) reported that heat stress changes the levels of inflammatory mediators secreted by glial cells. This induces neuroinflammation, impairing neurogenesis in the hippocampus and resulting in cognitive dysfunction. In a rat heatstroke model by Chauhan et al. (2021), increased levels of caspase 3 and Fluoro Jade-C were observed in the hippocampus, indicating apoptosis and neuronal damage in the hippocampus.

### 2.5 How can heatstroke damage the brain?

Research has documented that the brain is one of the organs most vulnerable to hyperthermia (Malamud et al., 1946). Heatstroke can lead to neurological complications, such as cerebellar ataxia, cognitive impairment, dysphagia, and aphasia (Leon and Bouchama, 2015). Long-term studies have revealed cellular damage in various brain regions, including the cerebellum, hippocampus, midbrain, and thalamus (Lawton et al., 2019). Hyperthermic stress, resulting from external temperature elevation and thermoregulation failure, leads to elevated temperature in brain tissues.

The blood–brain barrier (BBB) is a dynamic system for the exchange of substances between the blood and brain parenchyma and is an essential functional gatekeeper of the CNS (Hu and Tao, 2021). The BBB integrity is crucial for maintaining CNS homeostasis (Segarra et al., 2021). The tightness and integrity of the BBB vary in response to multiple factors, including environmental and systemic factors. Of these, extreme temperatures have been reported to affect BBB permeability (Kiyatkin and Sharma, 2009), with a decrease in brain blood supply and an increase in intracranial pressure resulting from a compromised BBB.

In addition to the direct effects of heat, we cannot ignore the indirect effects from multiple organs. A typical example is the intestinal tract. SIRS following hyperthermia damages intestinal epithelial cells, causing the relaxation of tight junctions, and bacteria and endotoxins from the intestinal tract enter the lymphatic flow (Gupta et al., 2017). This is referred to as bacterial translocation. Bacteria and endotoxins that invade the lymphatic stream act as danger signals (alermin) to the organism, which activate the inflammasome, the source of inflammation, via PRRs. The activated inflammasome activates proinflammatory cytokines such as IL-1β and IL-18 (Yin et al., 2022). These cytokines also increase vascular permeability in the CNS, leading to angiogenic and cytotoxic edema (Du et al., 2024). This enhanced leakage of proteins and pathogens from the systemic circulation to the brain leads to an inflammatory response that affects normal brain function in the chronic phase.

Heat also causes injury to vascular epithelial cells. The superficial layer of vascular endothelial cells contains a protective layer called glycocalyx. SIRS following hyperthermia damages this layer, allowing albumin and fluid components to leak out of the vessel into the interstitium, leading to edema of the interstitium (Kobayashi et al., 2018; Peng et al., 2023). Glycocalyx also plays a role in inhibiting intravascular thrombus formation, and its injury promotes intravascular thrombus formation. Both factors lead to blood flow injury (Peng et al., 2023). This also causes intracranial induction, resulting in a natural reduction of cerebral blood flow. Decreased cerebral blood flow leads to hypoxia of the brain parenchyma.

**Figure 1:**
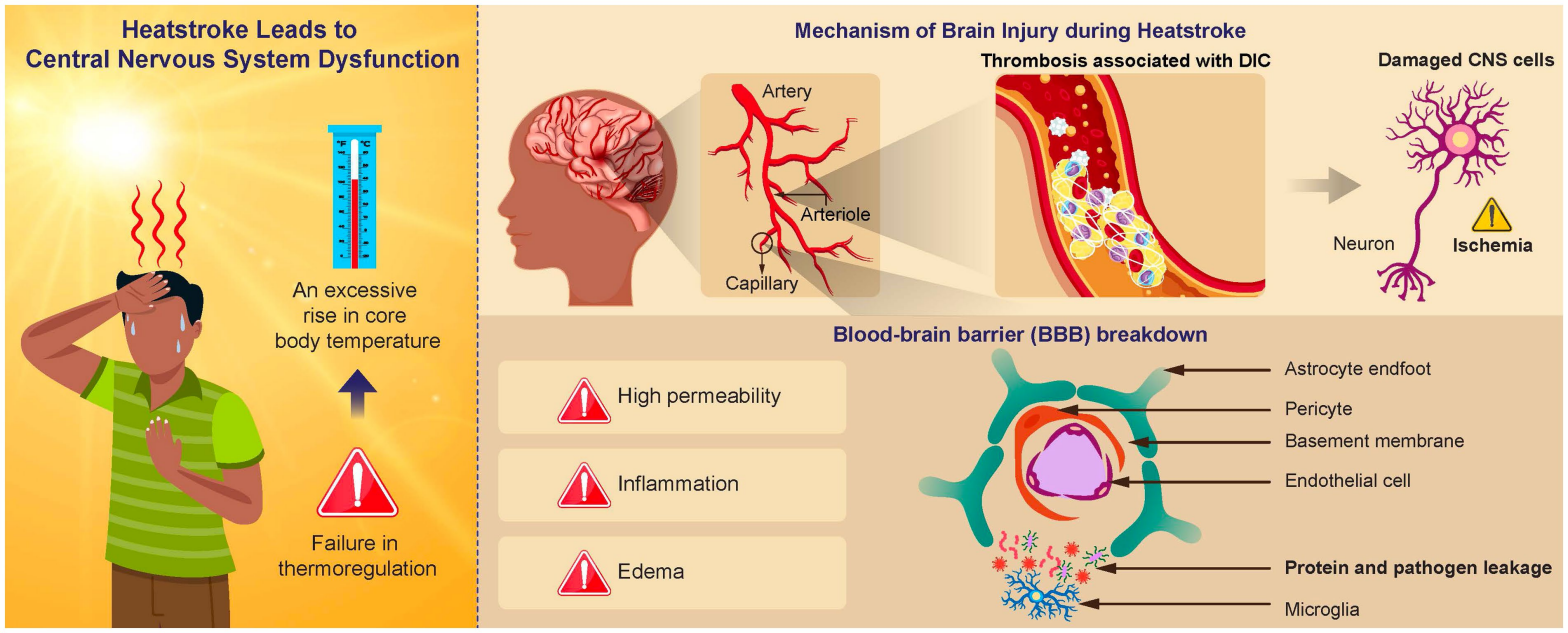
Mechanism of brain injury in heatstroke. An elevated brain temperature causes cell damage within the central nervous system (CNS) and disruption of the blood–brain barrier (BBB). Vasogenic and cytotoxic edema can result from increased BBB permeability, which facilitates protein and pathogen leakage from the systemic circulation into the brain. This leakage causes an inflammatory response, negatively affecting normal functioning. Activation of the inflammation and coagulation responses can result in the formation of immune thrombosis in the brain microvasculature, leading to an ischemic change in the CNS.

Thus, hyperthermia-associated SIRS leads to direct and indirect damage to the brain tissue, resulting in CNS symptoms such as impaired consciousness and coma (Figure 1).

### 2.6 Neuroimaging for heatstroke

The initial CT of patients suffering from heatstroke typically does not show any abnormalities (Albukrek et al., 1997) or only shows indirect signs of brain swelling (Szold et al., 2002). Previous MRI studies have identified lesions in different parts of the brain, including the external capsule, internal capsule, splenium, basal ganglia, thalamus, hippocampus, frontal and parietal cortex, insula, and subcortical white matter (McLaughlin et al., 2003; Sudhakar and Al-Hashimi 2007; Mahajan and Schucany, 2008; Lee et al., 2009; Fatih Yilmaz et al., 2018; Lee, 2020; Shimada et al., 2020). These lesions appear hypointense on T1-weighted sequences and hyperintense on T2-weighted, FLAIR, and DWI sequences. Most reported cases show cytotoxic swelling at different parts of the brain and cerebellar atrophy at 90 days post-heatstroke event (Szold et al., 2002; Mahajan and Schucany, 2008; Lee et al., 2009; Lee, 2020; Shimada et al., 2020). These findings suggest cerebral venous thrombosis, micro-level ischemia, and hemorrhagic processes related to heatstroke. Other imaging modalities include contrast-enhanced sequences that might reveal disruptions in the BBB due to vascular or inflammatory processes. Single-photon emission CT (SPECT) is excellent for evaluating brain hemodynamics. SPECT images are reported to be useful for predicting sequelae (Suzuki et al., 2023).

### 2.7 Gene expression and heatstroke

Several factors predispose to the development of heatstroke (Walter and Carraretto, 2015), among which genetic predisposition as an intrinsic cause of heatstroke is particularly notable. Conducting research to understand the detailed molecular pathogenesis of heat-related diseases and develop new diagnostic methods is crucial (Bouchama et al., 2022).

Recent advances in sequencing and other technologies have made it possible to comprehensively evaluate genes and gene expression using clinical specimens. Using these new techniques, heatstroke can be evaluated from the perspective of its molecular pathogenesis (Supplementary Table S3). For example, Oda et al. (2019) measured carnitine palmitoyltransferase II (CPT II), which is important for mitochondrial energy production, in patients with heatstroke and healthy controls. It is known that patients with a CPT II phenotype of [1,055 T > G/FF352C] cannot produce sufficient energy in a hyperthermic environment. It was also revealed that the prognosis of patients with severe heatstroke who exhibit this phenotype is poor (Oda et al., 2019). Identifying the expression of these genes by qRT-PCR may lead to the early detection of heatstroke and the development of therapeutic agents targeting specific molecules. Various genes related to heatstroke have been identified; however, those related to the prognosis of brain damage (e.g., biomarkers) require more research.

### 2.8 Role of biomarkers in predicting heatstroke neurological outcomes

Biomarkers play a crucial role in predicting the outcomes of patients with heatstroke by detecting early organ injury and guiding the development of novel therapeutic strategies for organ preservation. These biomarkers, obtained from biological samples of patients with heatstroke, help identify tissue damage, predict outcomes, and monitor recovery or repair failure. Various biomarkers have been identified in different organs affected by heatstroke, such as HMGB1 (Tong et al., 2011), plasma and urinary neutrophil gelatinase-associated lipocalin in the kidneys, intestinal fatty acid-binding protein 2 in the intestines, and creatine kinase and myoglobin in skeletal muscles (Schlader et al., 2022). Serum S100β and neuron-specific enolase (NSE) have attracted attention as CNS biomarkers (Supplementary Table S4) (Persson et al., 1987). S100β is a glial cell-specific protein expressed mainly in astrocytes and functions as a neurotrophic factor. S100β is secreted by damaged cells, with elevation in levels early after CNS injury (Papa et al., 2016). NSE is derived from neurons and is elevated in the blood following neuronal injury when the BBB is compromised. NSE is a protein of the myelin sheath and, thus, is elevated with myelin injury (Zhang et al., 2024). NSE is used as a biomarker for various neurological diseases, such as traumatic brain injury and stroke (Schlader et al., 2022). NSE is also gaining attention as a biomarker of heatstroke. First, it has been reported that S100β in serum and spinal fluid increases in heatstroke. A five-fold increase in serum S100β levels was reported in patients with a poor prognosis compared to those with a good prognosis (Chun et al., 2019), with the increase in S100β level resulting from impaired function of the BBB under high-temperature conditions (Watson et al., 2005). There is a strong correlation between NSE levels and neurological outcomes up to day seven after a heatstroke (Schlader et al., 2022). Myelin basic protein in the spinal fluid is also elevated in heatstroke owing to injury to the myelin sheath and the resulting neuronal damage with increasing core temperature. In contrast, the spinal and serum levels of IL-6, a marker of early inflammation, are typically low in heatstroke. This suggests that changes in the structural protein levels are a causative factor of heatstroke before the onset of inflammation (Ikeda et al., 2021). In addition, glial fibrillary acid protein and ubiquitin carboxyl-terminal hydrolase isozyme L1 are other known neural biomarkers; however, no studies have demonstrated their association with increased body temperature or neurological outcomes in heatstroke (Stacey et al., 2023). Further research is needed to translate these biomarkers into clinical practice and understand their utility in predicting long-term health outcomes following heatstroke.

### 2.9 Heatstroke and the cerebrospinal fluid (CSF)

The blood-cerebrospinal fluid barrier (BCSFB) in the choroid plexus cooperates with the cerebral blood barrier to regulate the CSF and stabilize the neuronal environment. Heat stress can damage the BCSFB, leading to cerebral edema and reduced cerebral blood flow, resulting in the leakage of various proteins and cytokines into the CSF (Sharma and Johanson, 2007; Hashim, 2010). As noted in section 2.8, S100β and NSE are the most frequently reported biomarkers associated with heatstroke and CSF abnormalities. They are elevated in nervous system cell damage as well as in patients with poor neurological prognosis (Persson et al., 1987; Frosini et al., 2000; Frosini, 2007; Chun et al., 2019; Li et al., 2020; Schlader et al., 2022). Although these markers can also be measured in serum, the CSF may reveal more subtle changes, with some reports indicating higher values in the CSF. Additionally, elevated levels of neurofilament light chain, suggesting axonal degeneration, as well as amino acids such as taurine and GABA, and inflammatory cytokines have been observed (Hashim, 2010; Lin et al., 2018; Gaetani et al., 2019; Azcue et al., 2024). While no studies directly compare the effectiveness of these CSF tests with other diagnostic methods, CSF testing offers advantages such as ease of sampling and earlier detection of changes compared to imaging tests. It also provides valuable insights into neurological prognosis; thus, selecting the appropriate test should consider the disease stage and specific clinical setting.

### 2.10 Management of heatstroke

The primary treatment for heatstroke involves rapid cooling to lower body temperature. Ice-water immersion is considered the gold standard for treating exertional heatstroke (Kim et al., 2020), particularly in young, fit individuals and has demonstrated a zero-fatality rate in large case series (Gaudio and Grissom, 2016). Other cooling methods include evaporative cooling using mist sprays and fans; Tarp-Assisted Cooling Oscillation (TACO) as an alternative when ice-water immersion is unavailable; strategic application of ice packs to cool the neck, axilla, and groin; cooling infusions; and intravascular and body surface cooling devices (Gaudio and Grissom, 2016; Luhring et al., 2016; Kim et al., 2020). The prompt initiation of cooling measures is crucial to prevent irreversible organ damage (Gaudio and Grissom, 2016). Supportive care is essential to manage complications and support organ function.

Techniques for cooling patients with hyperthermia are similar to those used for targeted temperature management after cardiac arrest or traumatic brain injury (Behr et al., 1997; Zeiner et al., 2001; Rossi et al., 2001; Wakino et al., 2005). These techniques include the administration of cold intravenous fluid; gastric, peritoneal, pleural, and/or bladder lavage with cold water; and the use of intravascular cooling techniques, such as cooling catheters and extracorporeal circuits (continuous venovenous hemofiltration or cardiopulmonary bypass). Extracorporeal membrane oxygenation, equipped with a blood cooling system, to achieve rapid whole-body cooling and brain cryotherapy for temperature control has been reported to be effective in treating abnormal hyperthermia and brain swelling accompanied by impaired consciousness in severe heatstroke (Fujita et al., 2021).

Using an intravascular cooling system has been reported as an active cooling treatment for heatstroke, and in cases where multiple factors contribute to the onset of sequelae and poor prognosis, invasive treatment should also be considered. However, simply normalizing body temperature may not be enough to fully prevent CNS damage, as it does not address inflammatory reactions, coagulation abnormalities, or vascular endothelial damage caused by excessive cytokines (Bouchama and Knochel, 2002). Previous animal experiments have shown a reduction in mortality and CNS damage with the administration of activated protein C (Chen et al., 2006; Brueckmann et al., 2006) and hyperbaric oxygen therapy for heatstroke (Tsai et al., 2005). In addition to active cooling treatment and systemic management, future treatment strategies should also focus on protecting the CNS.

## 3 Conclusion

In this mini review, we describe the clinical features, pathophysiology, and treatment of heatstroke. Additionally, we highlight the importance of understanding the underlying mechanisms of brain injury and identifying diagnostic biomarkers. Further research is needed to develop more effective treatment strategies and understand the long-term health consequences of heatstroke.

## References

[ref1] Albukrek D. Bakon M. Moran D. S. Faibel M. Epstein Y. (1997). Heat-stroke-induced cerebellar atrophy: clinical course, CT and MRI findings. Neuroradiology 39, 195–197. doi: 10.1007/s002340050392, PMID: 9106293

[ref2] Azcue N. Tijero-Merino B. Acera M. Pérez-Garay R. Fernández-Valle T. Ayo-Mentxakatorre N.. (2024). Plasma neurofilament light chain: a potential biomarker for neurological dysfunction in myalgic encephalomyelitis/chronic fatigue syndrome. Biomedicines 12:1539. doi: 10.3390/biomedicines12071539, PMID: 39062112 PMC11274366

[ref3] Barletta J. F. Palmieri T. L. Toomey S. A. Harrod C. G. Murthy S. Bailey H. (2024). Management of heat-related illness and injury in the ICU: a concise definitive review. Crit. Care Med. 52, 362–375. doi: 10.1097/CCM.0000000000006170, PMID: 38240487

[ref4] Bazille C. Megarbane B. Bensimhon D. Lavergne-Slove A. Baglin A. C. Loirat P.. (2005). Brain damage after heat stroke. J. Neuropathol. Exp. Neurol. 64, 970–975. doi: 10.1097/01.jnen.0000186924.88333.0d, PMID: 16254491

[ref5] Becker J. A. Stewart L. K. (2011). Heat-related illness. Am. Fam. Physician 83, 1325–1330, PMID: 21661715

[ref6] Behr R. Erlingspiel D. Becker A. (1997). Early and longtime modifications of temperature regulation after severe head injury. Prognostic implications. Ann. N. Y. Acad. Sci. 813, 722–732. doi: 10.1111/j.1749-6632.1997.tb51774.x, PMID: 9100962

[ref7] Bouchama A. Abuyassin B. Lehe C. Laitano O. Jay O. O’Connor F. G.. (2022). Classic and exertional heatstroke. Nat. Rev. Dis. Primers 8:8. doi: 10.1038/s41572-021-00334-6 35115565

[ref8] Bouchama A. Knochel J. P. (2002). Heat stroke. N. Engl. J. Med. 346, 1978–1988. doi: 10.1056/NEJMra011089, PMID: 12075060

[ref10] Brueckmann M. Hoffmann U. Borggrefe M. (2006). Beyond sepsis: activated protein C and heat stroke. Crit. Care Med. 34, 2020–2021. doi: 10.1097/01.CCM.0000221923.21401.71, PMID: 16801873

[ref11] Cao L. Wang J. Gao Y. Liang Y. Yan J. Zhang Y.. (2019). Magnetic resonance imaging and magnetic resonance venography features in heat stroke: a case report. BMC Neurol. 19:133. doi: 10.1186/s12883-019-1363-x 31215399 PMC6580543

[ref1002] Chauhan N. R. Kapoor M. Prabha Singh L. Gupta R. K. Chand Meena R. Tulsawani R.. (2017). Heat stress-induced neuroinflammation and aberration in monoamine levels in hypothalamus are associated with temperature dysregulation. Neuroscience. 358, 79–92. doi: 10.1016/j.neuroscience.2017.06.023, PMID: 28663093

[ref12] Chauhan N. R. Kumar R. Gupta A. Meena R. C. Nanda S. Mishra K. P.. (2021). Heat stress induced oxidative damage and perturbation in BDNF/ERK1/2/CREB axis in hippocampus impairs spatial memory. Behav. Brain Res. 396:112895. doi: 10.1016/j.bbr.2020.112895, PMID: 32890597

[ref13] Chen C.-M. Hou C.-C. Cheng K.-C. Tian R.-L. Chang C.-P. Lin M.-T. (2006). Activated protein C therapy in a rat heat stroke model. Crit. Care Med. 34, 1960–1966. doi: 10.1097/01.CCM.0000224231.01533.B1, PMID: 16715032

[ref14] Chun J.-K. Choi S. Kim H.-H. Yang H. W. Kim C. S. (2019). Predictors of poor prognosis in patients with heat stroke. Clin. Exp. Emerg. Med. 6, 345–350. doi: 10.15441/ceem.18.081, PMID: 31910506 PMC6952628

[ref15] D’Souza C. A. Rush S. J. Brown I. R. (1998). Effect of hyperthermia on the transcription rate of heat-shock genes in the rabbit cerebellum and retina assayed by nuclear run-ons. J. Neurosci. Res. 52, 538–548. doi: 10.1002/(SICI)1097-4547(19980601)52:5<538::AID-JNR6>3.0.CO;2-D, PMID: 9632310

[ref16] Dematte J. E. O’Mara K. Buescher J. Whitney C. G. Forsythe S. McNamee T.. (1998). Near-fatal heat stroke during the 1995 heat wave in Chicago. Ann. Intern. Med. 129, 173–181. doi: 10.7326/0003-4819-129-3-199808010-00001, PMID: 9696724

[ref17] Du G. Yang Z. Wen Y. Li X. Zhong W. Li Z.. (2024). Heat stress induces IL-1β and IL-18 overproduction via ROS-activated NLRP3 inflammasome: implication in neuroinflammation in mice with heat stroke. Neuroreport 35, 558–567. doi: 10.1097/WNR.0000000000002042, PMID: 38687900

[ref18] Epstein Y. Yanovich R. (2019). Heatstroke. N. Engl. J. Med. 380, 2449–2459. doi: 10.1056/NEJMra1810762, PMID: 31216400

[ref19] Fang W. Yin B. Fang Z. Tian M. Ke L. Ma X.. (2024). Heat stroke-induced cerebral cortex nerve injury by mitochondrial dysfunction: a comprehensive multi-omics profiling analysis. Sci. Total Environ. 919:170869. doi: 10.1016/j.scitotenv.2024.170869 38342446

[ref20] Fatih Yilmaz T. Aralasmak A. Toprak H. Guler S. Tuzun U. Alkan A. (2018). MRI and MR spectroscopy features of heat stroke: a case report. Iran. J. Radiol. 15:e62386. doi: 10.5812/iranjradiol.62386

[ref21] Frosini M. (2007). Changes in CSF composition during heat stress and fever in conscious rabbits. Prog. Brain Res. 162, 449–457. doi: 10.1016/S0079-6123(06)62022-0 17645932

[ref22] Frosini M. Sesti C. Palmi M. Valoti M. Fusi F. Mantovani P.. (2000). Heat-stress-induced hyperthermia alters CSF osmolality and composition in conscious rabbits. Am. J. Physiol. Regul. Integr. Comp. Physiol. 279, R2095–R2103. doi: 10.1152/ajpregu.2000.279.6.R2095, PMID: 11080074

[ref23] Fujita M. Miyazaki K. Horiguchi M. Yamamoto K. Ito S. Fukushima H. (2021). Veno-arterial extracorporeal membrane oxygenation for severe heatstroke with refractory hemodynamic failure. Case Rep. Acute Med. 4, 76–79. doi: 10.1159/000517681

[ref24] Gaetani L. Blennow K. Calabresi P. Di Filippo M. Parnetti L. Zetterberg H. (2019). Neurofilament light chain as a biomarker in neurological disorders. J. Neurol. Neurosurg. Psychiatry 90, 870–881. doi: 10.1136/jnnp-2018-320106, PMID: 30967444

[ref25] Gaudio F. G. Grissom C. K. (2016). Cooling methods in heat stroke. J. Emerg. Med. 50, 607–616. doi: 10.1016/j.jemermed.2015.09.014, PMID: 26525947

[ref26] Gupta A. Chauhan N. R. Chowdhury D. Singh A. Meena R. C. Chakrabarti A.. (2017). Heat stress modulated gastrointestinal barrier dysfunction: role of tight junctions and heat shock proteins. Scand. J. Gastroenterol. 52, 1315–1319. doi: 10.1080/00365521.2017.1377285, PMID: 28906161

[ref27] Hashim I. A. (2010). Clinical biochemistry of hyperthermia. Ann. Clin. Biochem. 47, 516–523. doi: 10.1258/acb.2010.010186, PMID: 20926467

[ref28] Hifumi T. Kondo Y. Shimizu K. Miyake Y. (2018). Heat stroke. J. Intensive Care 6:30. doi: 10.1186/s40560-018-0298-4 29850022 PMC5964884

[ref29] Hsiao S.-H. Chang C.-P. Chiu T.-H. Lin M.-T. (2007). Resuscitation from experimental heatstroke by brain cooling therapy. Resuscitation 73, 437–445. doi: 10.1016/j.resuscitation.2006.11.003, PMID: 17300862

[ref30] Hu Y. Tao W. (2021). Microenvironmental variations after blood-brain barrier breakdown in traumatic brain injury. Front. Mol. Neurosci. 14:750810. doi: 10.3389/fnmol.2021.750810, PMID: 34899180 PMC8662751

[ref31] Iba T. Connors J. M. Levi M. Levy J. H. (2022). Heatstroke-induced coagulopathy: biomarkers, mechanistic insights, and patient management. EClinicalMedicine 44:101276. doi: 10.1016/j.eclinm.2022.101276, PMID: 35128366 PMC8792067

[ref32] Ikeda T. Tani N. Watanabe M. Hirokawa T. Ikeda K. Morioka F.. (2021). Evaluation of cytokines and structural proteins to analyze the pathology of febrile central nervous system disease. Leg. Med. (Tokyo) 51:101864. doi: 10.1016/j.legalmed.2021.101864, PMID: 33798967

[ref33] Jung I. Choi S.-Y. Kim H.-J. Kim J.-S. (2017). Delayed vestibulopathy after heat exposure. J. Neurol. 264, 49–53. doi: 10.1007/s00415-016-8322-x, PMID: 27766472

[ref34] Kim D. A. Lindquist B. D. Shen S. H. Wagner A. M. Lipman G. S. (2020). A body bag can save your life: a novel method of cold water immersion for heat stroke treatment. J. Am. Coll. Emerg. Physicians Open 1, 49–52. doi: 10.1002/emp2.12007, PMID: 33000014 PMC7493529

[ref35] Kiyatkin E. A. Sharma H. S. (2009). Permeability of the blood-brain barrier depends on brain temperature. Neuroscience 161, 926–939. doi: 10.1016/j.neuroscience.2009.04.004, PMID: 19362131 PMC2694729

[ref36] Kobayashi K. Mimuro S. Sato T. Kobayashi A. Kawashima S. Makino H.. (2018). Dexmedetomidine preserves the endothelial glycocalyx and improves survival in a rat heatstroke model. J. Anesth. 32, 880–885. doi: 10.1007/s00540-018-2568-7, PMID: 30374889

[ref37] Lawton E. M. Pearce H. Gabb G. M. (2019). Review article: environmental heatstroke and long-term clinical neurological outcomes: a literature review of case reports and case series 2000–2016. Emerg. Med. Australas. 31, 163–173. doi: 10.1111/1742-6723.12990, PMID: 29851280

[ref38] Lee B. H. (2020). Atypical brain imaging findings associated with heat stroke: a patient with rhabdomyolysis and acute kidney injury: a case report. Radiol. Case Rep. 15, 560–563. doi: 10.1016/j.radcr.2020.02.007, PMID: 32292535 PMC7149585

[ref39] Lee J. S. Choi J. C. Kang S. Y. Kang J. H. Park J. K. (2009). Heat stroke: increased signal intensity in the bilateral cerebellar dentate nuclei and splenium on diffusion-weighted MR imaging. AJNR Am. J. Neuroradiol. 30:E58. doi: 10.3174/ajnr.A1432 19179428 PMC7051769

[ref40] Leon L. R. Bouchama A. (2015). Heat stroke. Compr. Physiol. 5, 611–647. doi: 10.1002/cphy.c140017, PMID: 25880507

[ref41] Leon L. R. Helwig B. G. (2010). Heat stroke: role of the systemic inflammatory response. J. Appl. Physiol. 109, 1980–1988. doi: 10.1152/japplphysiol.00301.2010, PMID: 20522730

[ref42] Li B. Jia Y.-R. Gao W. Li H.-P. Tao W.-H. Zhang H.-X. (2020). The expression and clinical significance of neuron specific enolase and S100B protein in patients of severe heatstroke-induced brain injury. Med. J. Chin. Peoples Liberation Army 45, 1282–1287.

[ref43] Li J. Zhang X.-Y. Wang B. Zou Z.-M. Wang P.-Y. Xia J.-K.. (2015). Diffusion tensor imaging of the cerebellum in patients after heat stroke. Acta Neurol. Belg. 115, 147–150. doi: 10.1007/s13760-014-0343-6, PMID: 25082094

[ref44] Lin Y.-F. Liu T.-T. Hu C.-H. Chen C.-C. Wang J.-Y. (2018). Expressions of chemokines and their receptors in the brain after heat stroke-induced cortical damage. J. Neuroimmunol. 318, 15–20. doi: 10.1016/j.jneuroim.2018.01.014, PMID: 29395321

[ref45] Liu W.-S. Chen C.-T. Foo N.-H. Huang H.-R. Wang J.-J. Chen S.-H.. (2009). Human umbilical cord blood cells protect against hypothalamic apoptosis and systemic inflammation response during heatstroke in rats. Pediatr. Neonatol. 50, 208–216. doi: 10.1016/S1875-9572(09)60065-6, PMID: 19856864

[ref46] Luhring K. E. Butts C. L. Smith C. R. Bonacci J. A. Ylanan R. C. Ganio M. S.. (2016). Cooling effectiveness of a modified cold-water immersion method after exercise-induced hyperthermia. J. Athl. Train. 51, 946–951. doi: 10.4085/1062-6050-51.12.07, PMID: 27874299 PMC5224736

[ref47] Mahajan S. Schucany W. G. (2008). Symmetric bilateral caudate, hippocampal, cerebellar, and subcortical white matter MRI abnormalities in an adult patient with heat stroke. Proc. (Bayl Univ Med Cent) 21, 433–436. doi: 10.1080/08998280.2008.11928446, PMID: 18982090 PMC2566920

[ref48] Malamud N. Haymaker W. Custer R. P. (1946). Heat stroke; a clinico-pathologic study of 125 fatal cases. Mil. Mil. Surg. 99, 397–449, PMID: 20276794

[ref49] McLaughlin C. T. Kane A. G. Auber A. E. (2003). MR imaging of heat stroke: external capsule and thalamic T1 shortening and cerebellar injury. AJNR Am. J. Neuroradiol. 24, 1372–1375, PMID: 12917130 PMC7973664

[ref50] Oda J. Yukioka T. Azuma K. Arai T. Chida J. Kido H. (2019). Endogenous genetic risk factor for serious heatstroke: the thermolabile phenotype of carnitine palmitoyltransferase II variant. Acute Med. Surg. 6, 25–29. doi: 10.1002/ams2.373, PMID: 30651994 PMC6328901

[ref51] Papa L. Brophy G. M. Welch R. D. Lewis L. M. Braga C. F. Tan C. N.. (2016). Time course and diagnostic accuracy of glial and neuronal blood biomarkers GFAP and UCH-L1 in a large cohort of trauma patients with and without mild traumatic brain injury. JAMA Neurol. 73, 551–560. doi: 10.1001/jamaneurol.2016.0039 27018834 PMC8805143

[ref52] Pechlaner C. Kaneider N. C. Djanani A. Sandhofer A. Schratzberger P. Patsch J. R. (2002). Antithrombin and near-fatal exertional heat stroke. Acta Med. Austriaca 29, 107–111. doi: 10.1046/j.1563-2571.2002.02016.x, PMID: 12168565

[ref53] Peng N. Geng Y. Ouyang J. Liu S. Yuan F. Wan Y.. (2023). Endothelial glycocalyx injury is involved in heatstroke-associated coagulopathy and protected by N-acetylcysteine. Front. Immunol. 14:1159195. doi: 10.3389/fimmu.2023.1159195 37350963 PMC10283401

[ref54] Persson L. Hårdemark H. G. Gustafsson J. Rundström G. Mendel-Hartvig I. Esscher T.. (1987). S-100 protein and neuron-specific enolase in cerebrospinal fluid and serum: markers of cell damage in human central nervous system. Stroke 18, 911–918. doi: 10.1161/01.STR.18.5.911, PMID: 3629651

[ref55] Robine J.-M. Cheung S. L. Le Roy S. Van Oyen H. Griffiths C. Michel J.-P.. (2008). Death toll exceeded 70,000 in Europe during the summer of 2003. C. R. Biol. 331, 171–178. doi: 10.1016/j.crvi.2007.12.001, PMID: 18241810

[ref56] Rossi S. Zanier E. R. Mauri I. Columbo A. Stocchetti N. (2001). Brain temperature, body core temperature, and intracranial pressure in acute cerebral damage. J. Neurol. Neurosurg. Psychiatry 71, 448–454. doi: 10.1136/jnnp.71.4.448, PMID: 11561026 PMC1763520

[ref57] Rublee C. Dresser C. Giudice C. Lemery J. Sorensen C. (2021). Evidence-based heatstroke management in the emergency department. West. J. Emerg. Med. 22, 186–195. doi: 10.5811/westjem.2020.11.49007, PMID: 33856299 PMC7972371

[ref58] Savioli G. Zanza C. Longhitano Y. Nardone A. Varesi A. Ceresa I. F.. (2022). Heat-related illness in emergency and critical care: recommendations for recognition and management with medico-legal considerations. Biomedicines 10:2542. doi: 10.3390/biomedicines10102542, PMID: 36289804 PMC9599879

[ref59] Schlader Z. J. Davis M. S. Bouchama A. (2022). Biomarkers of heatstroke-induced organ injury and repair. Exp. Physiol. 107, 1159–1171. doi: 10.1113/EP090142, PMID: 35654394 PMC9529995

[ref60] Segarra M. Aburto M. R. Acker-Palmer A. (2021). Blood-brain barrier dynamics to maintain brain homeostasis. Trends Neurosci. 44, 393–405. doi: 10.1016/j.tins.2020.12.002, PMID: 33423792

[ref61] Sharma H. S. Cervós-Navarro J. Dey P. K. (1991). Acute heat exposure causes cellular alteration in cerebral cortex of young rats. Neuroreport 2, 155–158. doi: 10.1097/00001756-199103000-00012, PMID: 1768859

[ref62] Sharma H. S. Johanson C. E. (2007). Blood-cerebrospinal fluid barrier in hyperthermia. Prog. Brain Res. 162, 459–478. doi: 10.1016/S0079-6123(06)62023-2 17645933

[ref63] Shimada T. Miyamoto N. Shimada Y. Watanabe M. Shimura H. Ueno Y.. (2020). Analysis of clinical symptoms and brain MRI of heat stroke: 2 case reports and a literature review. J. Stroke Cerebrovasc. Dis. 29:104511. doi: 10.1016/j.jstrokecerebrovasdis.2019.104511, PMID: 31784378

[ref64] Stacey M. J. Leckie T. Fitzpatrick D. Hodgson L. Barden A. Jenkins R.. (2023). Neurobiomarker and body temperature responses to recreational marathon running. J. Sci. Med. Sport 26, 566–573. doi: 10.1016/j.jsams.2023.09.011, PMID: 37777396

[ref65] Sudhakar P. J. Al-Hashimi H. (2007). Bilateral hippocampal hyperintensities: a new finding in MR imaging of heat stroke. Pediatr. Radiol. 37, 1289–1291. doi: 10.1007/s00247-007-0612-0, PMID: 17899057

[ref66] Suzuki K. Miyamoto K. Kanai T. Kurihara M. Kikuchi K. Harano K.. (2023). Single-photon emission computed tomography (SPECT) predicted neurological prognosis in heat stroke: a case report. Heliyon 9:e18285. doi: 10.1016/j.heliyon.2023.e18285, PMID: 37539227 PMC10393623

[ref67] Szold O. Reider-Groswasser I. I. Ben Abraham R. Aviram G. Segev Y. Biderman P.. (2002). Gray-white matter discrimination–a possible marker for brain damage in heat stroke? Eur. J. Radiol. 43, 1–5. doi: 10.1016/S0720-048X(01)00467-3, PMID: 12065113

[ref68] Tai P.-A. Chang C.-K. Niu K.-C. Lin M.-T. Chiu W.-T. Lin J.-W. (2021). Attenuation of heat-induced hypothalamic ischemia, inflammation, and damage by hyperbaric oxygen in rats. J. Neurotrauma 38, 1185–1192. doi: 10.1089/neu.2010.1323, PMID: 20578826

[ref69] Tong H.-S. Tang Y.-Q. Chen Y. Qiu J.-M. Wen Q. Su L. (2011). Early elevated HMGB1 level predicting the outcome in exertional heatstroke. J. Trauma 71, 808–814. doi: 10.1097/TA.0b013e318220b957, PMID: 21841514

[ref70] Tsai H.-M. Gao C.-J. Li W.-X. Lin M.-T. Niu K.-C. (2005). Resuscitation from experimental heatstroke by hyperbaric oxygen therapy. Crit. Care Med. 33, 813–818. doi: 10.1097/01.CCM.0000159193.42628.E8, PMID: 15818110

[ref71] Van Stavern G. P. Biousse V. Newman N. J. Leingang J. C. (2000). Downbeat nystagmus from heat stroke. J. Neurol. Neurosurg. Psychiatry 69, 403–404. doi: 10.1136/jnnp.69.3.403, PMID: 10991650 PMC1737113

[ref72] Wakino S. Hori S. Mimura T. Fujishima S. Hayashi K. Inamoto H.. (2005). Heat stroke with multiple organ failure treated with cold hemodialysis and cold continuous hemodiafiltration: a case report. Ther. Apher. Dial. 9, 423–428. doi: 10.1111/j.1744-9987.2005.00321.x, PMID: 16202019

[ref73] Walter E. Carraretto M. (2015). Drug-induced hyperthermia in critical care. J. Intensive Care Soc. 16, 306–311. doi: 10.1177/1751143715583502 28979436 PMC5606458

[ref74] Walter E. J. Carraretto M. (2016). The neurological and cognitive consequences of hyperthermia. Crit Care. 199. doi: 10.1186/s13054-016-1376-4 27411704 PMC4944502

[ref75] Watson P. Shirreffs S. M. Maughan R. J. (2005). Blood-brain barrier integrity may be threatened by exercise in a warm environment. Am. J. Physiol. Regul. Integr. Comp. Physiol. 288, R1689–R1694. doi: 10.1152/ajpregu.00676.2004, PMID: 15650123

[ref76] Yin H. Wu M. Lu Y. Wu X. Yu B. Chen R.. (2022). HMGB1-activatied NLRP3 inflammasome induces thrombocytopenia in heatstroke rat. PeerJ 10:e13799. doi: 10.7717/peerj.13799, PMID: 35945940 PMC9357367

[ref77] Zeiner A. Holzer M. Sterz F. Schörkhuber W. Eisenburger P. Havel C.. (2001). Hyperthermia after cardiac arrest is associated with an unfavorable neurologic outcome. Arch. Intern. Med. 161, 2007–2012. doi: 10.1001/archinte.161.16.2007, PMID: 11525703

[ref78] Zhang Y. Li Z. Wang H. Pei Z. Zhao S. (2024). Molecular biomarkers of diffuse axonal injury: recent advances and future perspectives. Expert. Rev. Mol. Diagn. 24, 39–47. doi: 10.1080/14737159.2024.2303319, PMID: 38183228

[ref79] Zhu X. Huang J. Wu Y. Zhao S. Chai X. (2023). Effect of heat stress on hippocampal neurogenesis: insights into the cellular and molecular basis of neuroinflammation-induced deficits. Cell. Mol. Neurobiol. 43, 1–13. doi: 10.1007/s10571-021-01165-5, PMID: 34767143 PMC11415162

