# Supplementary material for: How can heatstroke damage the brain? A mini review
Source: Front Neurosci. 2024 Oct 10;18:1437216. doi: 10.3389/fnins.2024.1437216 (PMC11499184; doi:10.3389/fnins.2024.1437216)
Supplement: Supplementary file 1 [file Table_1.DOCX]

Supplementary Material

# Supplementary tables

**Supplemental Table 1: Worldwide Epidemiology of Heat Stroke**

| **Region** | **Mortality**  **(per 100,000)** | **Risk Factors** | **Key Observations** | **Reference** |
| --- | --- | --- | --- | --- |
| North America | 3.00-8.9 | Extreme heat waves, urban heat islands | Increased incidences in urban areas due to the heat island effect; southern regions most affected | Fuhrmann et al., 2016,  CDC, 2006 |
| Europe | 11.4 | Heatwaves, lack of acclimatization | Rising trends due to climate change, with Southern Europe at greater risk | Ballester et al., 2023 |
| Asia | 3.25-11 | High temperatures, humidity, urbanization | Notable incidences in South and Southeast Asia; rural areas particularly vulnerable | Burkart et al., 2021 |
| Africa | 0.09-1.68 | High temperatures, lack of infrastructure | Northern and sub-Saharan Africa face high risks; healthcare accessibility issues exacerbate outcomes | Hajat et a., 2023 |
| Australia | 0.04-0.65 | Heat waves, outdoor activities | Higher incidences in tropical northern regions; public health campaigns have mitigated risks | Coates et al., 2022 |
| South America | 0.03-2.71 | Climate variability, urban heat islands | Significant risks in the Amazon and coastal regions; socio-economic status affects susceptibility | CDC, 2006 |

Fuhrmann, C.M., Sugg, M.M., Konrad, C.E., and Waller, A. (2016) Impact of extreme heat events on emergency department visits in North Carolina (2007–2011). J. Comm. Health. 41, 146–156. doi: 10.1007/s10900-015-0080-7.

Centers for Disease Control and Prevention (CDC) (2006) Heat-related deaths-United States, 1999-2003. MMWR Morb. Mortal Wkly. Rep. 55, 796–798.

Ballester, J., Quijal-Zamorano, M., Méndez Turrubiates, R.F., Pegenaute, F., Herrmann, F.R., Robine, J.M., et al. (2023) Heat-related mortality in Europe during the summer of 2022. Nat. Med. 29, 1857–1866. doi: [10.1038/s41591-023-02419-z](https://doi.org/10.1038/s41591-023-02419-z).

Burkart, K.G., Brauer, M., Aravkin, A.Y., Godwin, W.W., Hay, S.I., He, J., et al. (2021) Estimating the cause-specific relative risks of non-optimal temperature on daily mortality: a two-part modelling approach applied to the Global Burden of Disease Study. Lancet. 398, 685–697. doi: 10.1016/S0140-6736(21)01700-1. Erratum in Lancet (2021) 398, 685–697. doi: 10.1016/S0140-6736(21)01700-1.

Hajat, S., Proestos, Y., Araya-Lopez, J.L., Economou, T., and Lelieveld, J. (2023) Current and future trends in heat-related mortality in the MENA region: a health impact assessment with bias-adjusted statistically downscaled CMIP6 (SSP-based) data and Bayesian inference. Lancet. Planet. Health. 7, e282–e290. doi: 10.1016/S2542-5196(23)00045-1

Coates, L., van Leeuwen, J., Browning, S., Gissing, A., Bratchell, J., and Avci, A. (2022) Heatwave fatalities in Australia, 2001–2018: An analysis of coronial records. Int. J. Disaster. Risk. Reduc. 102671, 67. doi: [10.1016/j.ijdrr.2021.102671](https://doi.org/10.1016/j.ijdrr.2021.102671).

**Supplemental Table 2. The 2015 Classification Criteria for Heat-Related Illnesses of the Japanese Association of Acute Medicine**

|  | | |
| --- | --- | --- |
| Classification based on clinical presentations | Symptoms | Treatment |
| **Heat-related cramp Heat-related syncope** | Dizziness, faintness, slight yawning Heavy sweating Muscle pain, stiff muscles (muscle cramps) No impairment in consciousness | **May be handled on site under normal conditions:** Rest in a cool environment, cooling of the body surface, and rehydration – oral water and Na+ |
| **Heat exhaustion** | Headache, vomiting, fatigue, clammy skin, and declining concentration and judgement | **Examination at a medical institution is necessary:** Body temperature management, rest in a cool environment, and rehydration –water and Na+ via intravenous drip if oral intake is difficult |
| **Heat stroke** | Includes at least one of the following: central nervous system manifestation (impaired consciousness, cerebellar symptoms, convulsive seizures), hepatic/renal dysfunction (requiring inpatient hospital care)  Coagulation disorder, according to acute phase of the DIC diagnostic criteria: most severe of the three types | **In-patient hospital care, including intensive care if necessary:** Body temperature management (internal body cooling and intravascular cooling, in combination with body surface cooling) Respiratory and circulatory care DIC treatment |

DIC, disseminated intravascular coagulation; JCS, Japan Coma Scale

**Supplemental Table 3. Gene expression changes associated with heatstroke**

| **SNP** | **Gene** | **Pathogen** | **Species** | **Samples** | **Reference** |
| --- | --- | --- | --- | --- | --- |
| 175A>G | Calsequestrin-1 (CASQ1) | Dysfunction of CASQ1 in calcium regulation during heatstroke | Human | Blood | Li et al., 2014 |
| 1055T>G/FF352C | Carnitine palmitoyltransferase (CPT) II | Dysfunction of CPT II in Mitochondrial energy production | Human | Blood | Oda et al., 2018 |
| **DNA** | **Gene** | **Pathogen** | **Species** | **Samples** | **Reference** |
|  | HSP60 gene | Facilitates the folding of peptides with adenosine triphosphate (ATP) and cofactor chaperonin-10; mediates protein importation into yeast mitochondria | Human | Tumor–tissue or blood | Hu et al., 2022 |
|  | HSP70 gene | Maintains the dynamic balance of the synthesis, folding, degradation, and translocation of proteins | Human | Tumor–tissue or blood | Hu et al., 2022 |
|  | HSP90 gene | Associated with steroid receptors and Src kinase | Mouse | Tumor xenografts | Niu et al., 2021 |
|  | HSP90 gene | Associated with steroid receptors and Src kinase | Human | Blood | Hu et al., 2022 |
|  | I-FABP gene | Evidence of increased intestinal permeability and/or injury | Human | Intestinal mucosa | Schlader et al., 2022 |
| **RNA** | **Gene** | **Pathogen** | **Species** | **Samples** | **Reference** |
|  | Exosomal miR-548x-3p | Inhibits pyroptosis of vascular endothelial cells through HMGB1 | Human | Blood | Pei et al., 2023 |
|  | GSDMD siRNA | Induced by heatstroke | Human | Umbilical vein endothelial cells (HUVECs) | Pei et al., 2018 |
|  | Microglial exosomal miR-466i-5p | Induces brain injury | Mouse | Heat-stressed neuronal cellular model | Zhu et al., 2022 |
|  | Heat shock protein transcription factor 1 (HSF1) | Master regulator of the heat shock genes | Chicken | Heart, liver, and breast muscles | Xie et al., 2014 |
|  | Heat shock protein transcription factor 2 (HSF2) | Participates in HSF1-mediated HSP expression | Chicken | Heart, liver, and breast muscles | Xie et al., 2014 |
|  | Heat shock protein transcription factor 3 (HSF3) | One of the main HSFs for heat shock response, with HSF1 activated at lower temperatures and binding with DNA | Chicken | Heart, liver, and breast muscles | Xie et al., 2014 |
|  | Heat shock protein transcription factor 4 (HSF4) | Exhibits tissue-specific expression and may function to repress the expression of genes encoding heat shock proteins and molecular chaperones | Chicken | Heart, liver, and breast muscles | Xie et al., 2014 |
|  | miR-511-3p, miR-122-5p, miR-155-3p, miR-1290, and let7-5p, whereas the most downregulated ones included miR-150-3p, 146a-5p, and 151a-3p | miRNAs of patients with heatstroke compared to control group were associated mostly with inflammatory response, including T cell activation, B cell receptor signaling, dendritic cell chemotaxis and leukocyte migration, and platelet activation and blood coagulation | Human | Blood | Li et al., 2021 |
|  | HSR | Encodes the known heat-inducible HSPs | Human | Blood | Bouchama et al., 2023 |
|  | BAG2 | Cochaperone and chaperonin genes | Human | Blood | Bouchama et al., 2023 |
|  | HSP60 | Cochaperone and chaperonin genes | Human | Blood | Bouchama et al., 2023 |
|  | TRiC (ring complex of the T complex protein) | Ring complex of the T complex protein | Human | Blood | Bouchama et al., 2023 |

Bouchama, A, Rashid, M., Malik, S.S., Al Mahri, S., Yassin, Y., Abdullah, M., et al. (2023) Whole genome transcriptomic reveals heat stroke molecular signatures in humans. J. Physiol. 601, 2407–2423. doi: 10.1113/JP284031.

Hu, C., Yang, J., Qi, Z., Wu, H., Wang, B., Zou, F., et al. (2022) Heat shock proteins: biological functions, pathological roles, and therapeutic opportunities. Med. Comm. 3, e161. doi: [10.1002/mco2.161](https://doi.org/10.1002/mco2.161).

Li, Y., Wang, Y., and Ma, L. (2014) An association study of CASQ1 gene polymorphisms and heat stroke. GPB 12, 127–132. doi: 10.1016/j.gpb.2014.03.004

Li, Y., Wen, Q., Chen, H., Wu, X., Liu, B., Li, H., et al. (2021) Exosomes derived from heat stroke cases carry miRNAs associated with inflammation and coagulation cascade. Front. Immunol. 12, 624753. doi: 10.3389/fimmu.2021.624753

Niu, M., Zhang, B., Li, L., Su, Z., Pu, W., Zhao, C., et al. (2021) Targeting HSP90 Iinhibits Proliferation proliferation and Induces induces Apoptosis apoptosis Through through AKT1/ERK Pathway pathway in Lung lung Cancercancer. Front. Pharmacol. 12, 724192. doi: 10.3389/fphar.2021.724192.

Oda, J., Yukioka, T., Azuma, K., Arai, T., Chida, J., and Kido, H. (2018) Endogenous genetic risk factor for serious heatstroke: the thermolabile phenotype of carnitine palmitoyltransferase II variant. Acute Med. Surg. 6, 25–29. doi: 10.1002/ams2.373.

Pei, Y., Geng, Y., Su, L., and Su, L. (2018) Pyroptosis of HUVECs can be induced by heat stroke. Biochem. Biophys. Res. Commun. 506, 626–631. doi: 10.1016/j.bbrc.2018.10.051.

Pei, Y., Ma, W., Wang, H., Chan, F., Xiao, W., Fan, M., et al. (2023) Mesenchymal stem cell-derived exosomal miR-548x-3p inhibits pyroptosis of vascular endothelial cells through HMGB1 in heat stroke. Genomics 115, 110719. doi: 10.1016/j.ygeno.2023.110719.

Schlader, Z.J., Davis, M.S., and Bouchama, A. (2022) Biomarkers of heatstroke-induced organ injury and repair. Exp. Physiol. 107, 1159–1171. doi: 10.1113/EP090142

Xie, J., Tang, L., Lu, L., Zhang, L., Xi, L., Liu, H.C., et al. (2014) Differential expression of heat shock transcription factors and heat shock proteins after acute and chronic heat stress in laying chickens (Gallus gallus). PLoS One 9, e102204. doi: 10.1371/journal.pone.0102204

Zhu, J., Chen, Y., Ji, J., Wang, L., Xie, G., Tang, Z., et al. (2022) Microglial exosomal miR-466i-5p induces brain injury via promoting hippocampal neuron apoptosis in heatstroke. Front. Immunol. 13, 968520. doi: 10.3389/fimmu.2022.968520.

**Supplemental Table 4. Biomarkers of Interest for Neurological Outcomes of a Heatstroke**

| **Biomarker** | **Locations of expression** | **Characteristics** | **Reference** |
| --- | --- | --- | --- |
| NSE | Neurons | NSE values are associated with neurological outcome up to day 7 after heatstroke. | Schlader et al., 2022 |
| S100β | Glial cells | S100β in serum and spinal fluid is elevated in heatstroke. | Chun et al., 2019 |
|  |  | Serum values are higher in patients with a poor prognosis at discharge compared with those with a good prognosis after a heatstroke | Chun et al., 2019 |
| MBP | Myelin | MBP values in spinal fluid are elevated. | Ikeda et al., 2021 |

NSE, neuron specific enolase; MBP, myelin basic protein

Chun, J.K., Choi, S., Kim, H.H., Yang, H.W., and Kim C.S. (2019) Predictors of poor prognosis in patients with heat stroke. Clin. Exp. Emerg. Med. 4, 345–350. doi: 10.15441/ceem.18.081.

Ikeda, T., Tani, N., Watanabe, M., Hirokawa, T., Ikeda, K., Moriaka, F., et al. (2021) Evaluation of cytokines and structural proteins to analyze the pathology of febrile central nervous system disease. Leg. Med. 51, 101864. doi. 10.1016/j.legalmed.2021.101864.

Schlader, Z.J., Davis, M.S., and Bouchama, A. (2022) Biomarkers of heatstroke-induced organ injury and repair. Exp. Physiol. 107, 1159–1171. doi: [10.1113/EP090142](https://doi.org/10.1113/ep090142)
